# Portable RF-Sensor System for the Monitoring of Air Pollution and Water Contamination

**DOI:** 10.1155/2012/568974

**Published:** 2012-08-15

**Authors:** Joonhee Kang, Jin Young Kim

**Affiliations:** ^1^Department of Physics, University of Incheon, Incheon 402-749, Republic of Korea; ^2^Intelligent Sensors Convergence Research Center, University of Incheon, Incheon 402-749, Republic of Korea

## Abstract

Monitoring air pollution including the contents of VOC, O_3_, NO_2_, and dusts has attracted a lot of interest in addition to the monitoring of water contamination because it affects directly to the quality of living conditions. Most of the current air pollution monitoring stations use the expensive and bulky instruments and are only installed in the very limited area. To bring the information of the air and water quality to the public in real time, it is important to construct portable monitoring systems and distribute them close to our everyday living places. In this work, we have constructed a low-cost portable RF sensor system by using 400 MHz transceiver to achieve this goal. Accuracy of the measurement was comparable to the ones used in the expensive and bulky commercial air pollution forecast systems.

## 1. Introduction

As modern technology advances rapidly, the environmental pollution has been also progressing very fast. As much as the development of technology has satisfied many people on their basic living conditions on foods, clothes, and dwellings, people became more aware of the quality of living conditions and started investing to achieve the clean environment.

The simplest method to monitor the deterioration of the environment is to sample the air, the soil, or the water from the contaminated area and to analyze them in the laboratory. But this method often takes too much time and efforts in sampling and testing. To solve this problem, a vehicle equipped with a test system can be sent to the contaminated area. However, an expensive special vehicle is needed, and this solution covers only quite limited region.

Due to the recent advancement in the various sensors and communication technologies, various monitoring stations could be constructed in the designated buildings or places to monitor the environmental contaminations. The measured data could be sent to the central server and processed to be announced to the public through the internet and through various media. By locating many stations in the wide area and employing advanced communication technology, effective monitoring of the environmental contamination may be achieved. However, establishing a monitoring station needs acquiring expensive equipment and a space to install bulky equipments. Also, the area covered by each station is limited only near its location. The better approach to achieve the effective monitoring is to develop a portable monitoring system by using a compact sensor system. In this work, we developed a low-cost portable RF sensor system to measure the air pollution and water contamination in addition to the real-time location information [1–5]. Our work contains the original concept to add the location information to the environmental pollution information collected from our scientifically engineered portable system.

## 2. System Design

We constructed a low-cost portable monitoring system by combining sensor unit, central processing unit, power unit, GPS unit, and RF unit. The sensor unit was to measure the air and water quality, the central processing unit for data acquisition and processing, the GPS unit to track the position of the RF-sensor in real time and the RF unit for the wireless transmission and reception of the data. The location information obtained with GPS unit was important to determine the polluted area and to manage the distribution of the sensor units.

**Figure 1:**
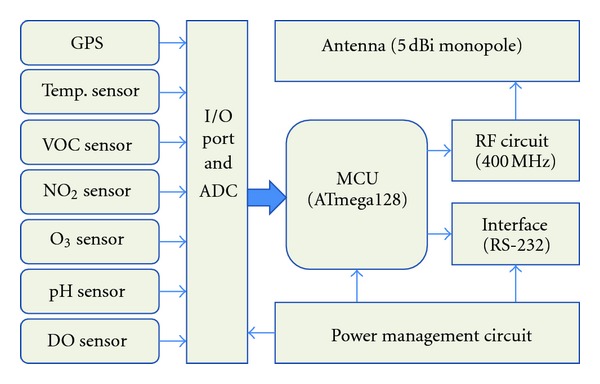
Block diagram of the RF sensor unit.

Figure 1 shows the block diagram of the RF sensor unit where various sensors (VOC, O_3_, NO_2_, pH, DO, and dust sensors), GPS and wireless transceiver circuits were included. DO sensor measures the dissolved oxygen content (DO value) and is widely used to determine the quality of water, along with the pH level measurement. The information from the sensor units was collected from the RF receiver and relayed to the control tower. As could be seen in Figure 1, the sensor unit includes MCU, power circuits, and RF transceiver circuits for wireless communication [6, 7].

In this work, we chose the low-cost portable sensors to apply to the air pollution and water contamination measurements. The choice of VOC; O_3_; NO_2_ gas sensors and pH; DO sensors were chosen because these were the most common air pollutants in Nam-dong industrial area in Incheon. Since the concentrations of the small factories were very dense in this area, we devised the portable system to effectively measure the pollutants. RF technology was used to conveniently collect the measured data in this sloppy area.

To monitor various kinds of air pollution we installed VOC, O_3_, NO_2_, and dust sensors in the unit. These various sensors had their own I/O ports to transfer the measured data to MCU. MCU had internal 12 bit ADC to convert the sensor output values to the digital values. The final processed information is transferred to the RF transceiver for data transmission. Remotely located central server will collect these data and use them for further process [8].

As we used small-size RF chips in our RF sensor system, the size of the system became remarkably smaller than the system used in the moving vehicle test station and the system used in the designated station. Also, our effective design reduced the cost of the system by an order of magnitude. Low cost and compactness of the system may allow us to install the systems in more densely populated locations and also to use the system in portable applications. The sensors employed in this system had good enough specifications to be used in the air pollution forecast systems. We summarized the hardware specifications of the RF sensor system in Table 1.

## 3. System Construction

**Figure 2:**
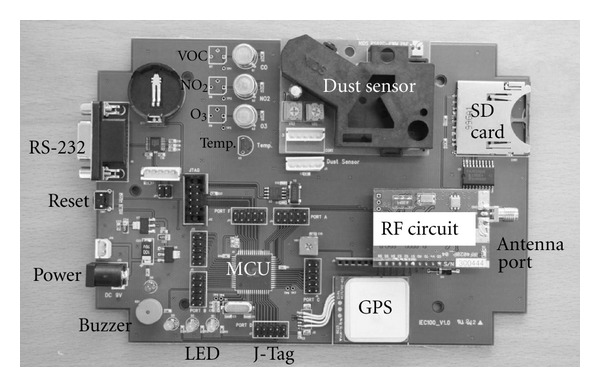
Picture of the completed air monitoring RF sensor unit.

Figure 2 shows the picture of the fabricated hardware of the RF sensor unit. As can be seen in the picture, the main parts of the constructed hardware were the interfaces, the power, the MCU, and the sensors part. Various sensors could be installed in the sensors part, and the extension of this part to include more sensors could be done very easily. The usual commercial products may use the pumps to collect the gases in the air and analyze them using the sensors. We could easily add the minipumps to our portable system. However, we obtained reasonably good results by directly exposing our unit to the contaminated air and concluded that our approach is good enough in the polluted environmental area. We gained the system performance from this approach in the size, the cost, and the power consumption.

We also installed SD memory card to save the data in case the data get lost during wireless data communications. In this way, we could obtain the reliable data and use the current design for further development to the commercial package. We used 400 MHz RF transceiver to achieve the moderate communication distance of 1 km. The installed GPS provided us with the current location information in real time while the sensors detect various contamination elements.

**Figure 3:**
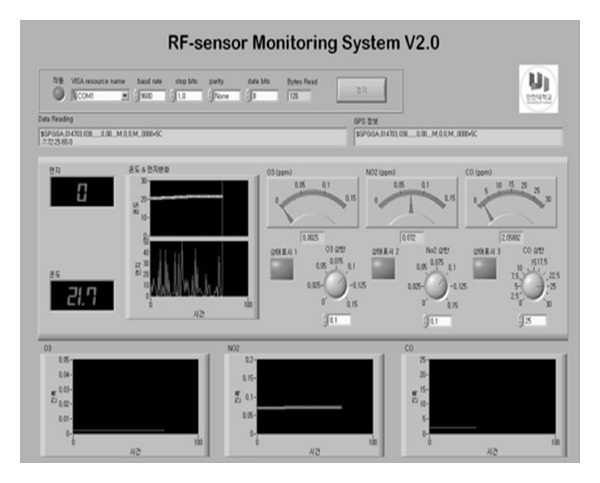
Virtual instrument constructed for our RF monitoring system.

Figure 3 shows the captured screen of the virtual instrument developed to control and monitor the RF sensor system. Data obtained from various sensors were displayed on the screen, and the status of the sensor unit could be monitored on the screen. We used Labview software from National Instruments [9–11].

## 4. Measurements

**Figure 4:**
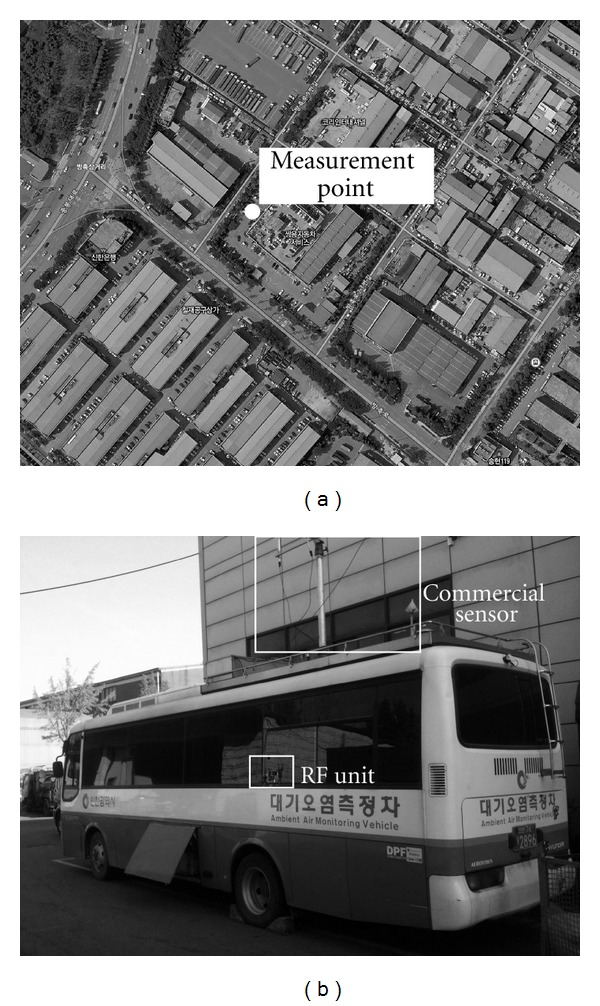
Map of the test location (a) and the picture of the air monitoring bus (b).

Figure 4 shows the map of the tested location and the moving vehicle where our sensor unit was installed and operated. RF unit could be seen on the bus window. In this work, we measured the air quality in Songrim-dong, Seo-gu, Incheon South Korea. We compared our data with the data obtained from already equipped bulky and expensive commercial equipment in the specialty bus.

**Figure 5:**
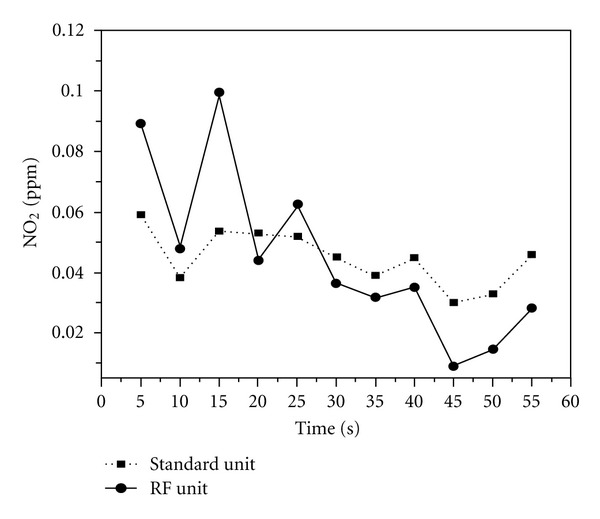
NO_2_ gas detection results for the portable RF-sensor unit and the commercial standard unit.

Figure 5 shows the NO_2_ gas detection results from our portable RF sensor unit. In Figure 5, we also presented the NO_2_ gas detection results obtained from the standard commercial unit. The maximum value measured with our unit was 0.1 ppm, and the minimum value was 0.01 ppm. The measurement was done over the time period of 1 hour. When we compared the average values from the two units, the average measured value was 0.045 ppm for our portable RF sensor system and 0.045 ppm from the commercial standard unit, resulting to the same value. Data collection rate was 2 readings/sec. Therefore, each measurement was from the average of 150 measurements. They were very reproducible.

**Figure 6:**
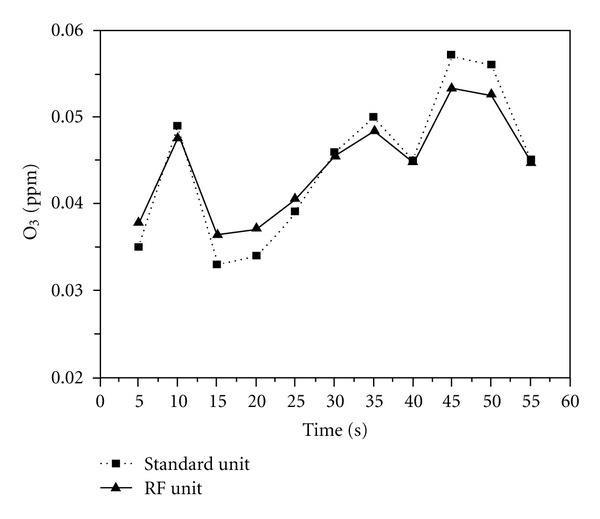
O_3_ gas detection results for the portable RF-sensor unit and the commercial standard unit.

Figure 6 shows the O_3_ gas detection results from our portable RF sensor unit. In Figure 6, we also presented the O_3_ gas detection results obtained with the standard commercial unit. Compared to the NO_2_ gas detection results, the values from the two units agreed much better. The maximum value measured with our unit was 0.05 ppm, and the minimum value was 0.037 ppm. The average value measured over 1 hour was 0.044 ppm for the portable RF sensor unit and 0.044 ppm for the commercial standard unit, resulting in the same value. Data collection rate was 2 readings/sec. Therefore, each measurement was from the average of 150 measurements. They were very reproducible.

**Figure 7:**
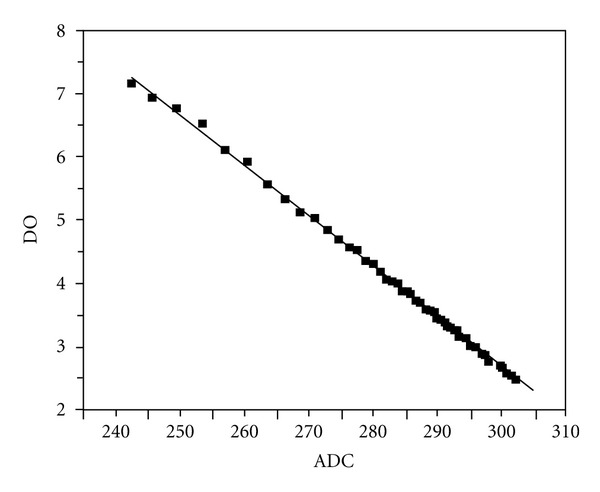
DO measurements.

**Figure 8:**
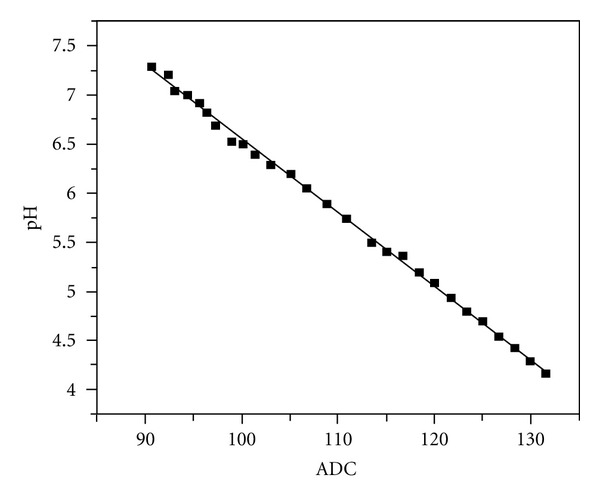
pH measurements.

Figures 7 and 8 show the test results obtained from the calibration measurements of DO and pH sensors. For pH test, we used buffer solution standard from Shinyo Pure Chemicals Co., Ltd. The horizontal axes represent the digital values corresponding to pH and DO values. These digital values were transferred to the MCU for any further processing of the data. In this experiment, these digital data were converted to pH values and DO values according to the linear fitting lines in the figures. Our system is in commercial use to monitor a water reservoir now.

## 5. Conclusion

We have designed portable RF sensor system to monitor the air pollution by measuring the contents of VOC, O_3_, NO_2_, and dusts. It also was used to monitor the water contamination by measuring the pH and DO of water. We also fabricated and tested the system. The performance tests of the unit were done for the O_3_ and NO_2_ gases and compared to the commercial standard unit used to forecast the air pollution status by the city of Incheon in South Korea. Accuracy of the measurements from our portable RF-sensor system was comparable to the commercial standard unit. Establishing monitoring stations can cost 100,000–200,000 US$ per station. However, our portable unit may cost less than only 2,000 US$ per unit. The low cost and the adapted wireless communication may enable to construct more dense air pollution monitoring network. The performance of our system may be enhanced by simply installing higher-performance sensors.

## Figures and Tables

**Table 1:** Specifications of the hardware and the sensors.

Size	170 × 120 mm^2^
MCU	ATmega128
Frequency	400 MHz
Antenna	5 dBi monopole
Power	9 V DC
VOC sensitivity	10–1000 ppm
O_3_ sensitivity	0.01–1 ppm
NO_2_ sensitivity	0.02–5 ppm
